# Association between the Number of Injuries Sustained and 12-Month Disability Outcomes: Evidence from the Injury-VIBES Study

**DOI:** 10.1371/journal.pone.0113467

**Published:** 2014-12-11

**Authors:** Belinda J. Gabbe, Pam M. Simpson, Ronan A. Lyons, Shanthi Ameratunga, James E. Harrison, Sarah Derrett, Suzanne Polinder, Gabrielle Davie, Frederick P. Rivara

**Affiliations:** 1 Department of Epidemiology and Preventive Medicine, Monash University, Melbourne, Australia; 2 Centre for Improvement of Population Health through E-records Research, Swansea University, Swansea, United Kingdom; 3 Public Health Wales NHS Trust, Cardiff, United Kingdom; 4 Section of Epidemiology and Biostatistics, School of Population Health, University of Auckland, Auckland, New Zealand; 5 Research Centre for Injury Studies, Flinders University, Adelaide, South Australia, Australia; 6 Injury Prevention Research Unit, Department of Preventive and Social Medicine, Dunedin School of Medicine, University of Otago, Dunedin, New Zealand; 7 School of Health and Social Services, College of Health, Massey University, Palmerston North, New Zealand; 8 Department of Public Health, Erasmus MC, Rotterdam, The Netherlands; 9 Departments of Pediatrics and Epidemiology, University of Washington, Seattle, Washington, United States of America; Texas Tech University Health Science Centers, United States of America

## Abstract

**Objective:**

To determine associations between the number of injuries sustained and three measures of disability 12-months post-injury for hospitalised patients.

**Methods:**

Data from 27,840 adult (18+ years) participants, hospitalised for injury, were extracted for analysis from the Validating and Improving injury Burden Estimates (Injury-VIBES) Study. Modified Poisson and linear regression analyses were used to estimate relative risks and mean differences, respectively, for a range of outcomes (Glasgow Outcome Scale-Extended, GOS-E; EQ-5D and 12-item Short Form health survey physical and mental component summary scores, PCS-12 and MCS-12) according to the number of injuries sustained, adjusted for age, sex and contributing study.

**Findings:**

More than half (54%) of patients had an injury to more than one ICD-10 body region and 62% had sustained more than one Global Burden of Disease injury type. The adjusted relative risk of a poor functional recovery (GOS-E<7) and of reporting problems on each of the items of the EQ-5D increased by 5–10% for each additional injury type, or body region, injured. Adjusted mean PCS-12 and MCS-12 scores worsened with each additional injury type, or body region, injured by 1.3–1.5 points and 0.5 points, respectively.

**Conclusions:**

Consistent and strong relationships exist between the number of injury types and body regions injured and 12-month functional and health status outcomes. Existing composite measures of anatomical injury severity such as the NISS or ISS, which use up to three diagnoses only, may be insufficient for characterising or accounting for multiple injuries in disability studies. Future studies should consider the impact of multiple injuries to avoid under-estimation of injury burden.

## Introduction

More than one injury can be sustained in a single event. Concurrent multiple injuries increase the risk of mortality, and the need for timely and multidisciplinary care for multiple injured patients is well defined [1]. While there is obvious potential for multiple injuries to increase the risk of long-term and permanent disability, the relationship between multiple injuries and disability outcomes has not been clearly established.

Numerous studies have investigated the association between anatomical measures of injury severity such as the Injury Severity Score (ISS) or New Injury Severity Score (NISS), which combine up to three injuries into a composite measure of severity based on a “threat to life” scale attributed to each diagnosis, and measures of longer term disability following injury [2], [3], [4], [5], [6]. Meerding et al investigated the number of injuries as a predictor of longer term function but their approach was limited to up to three recorded injuries [7]. Previous burden of disease studies have almost exclusively used the principal diagnosis or “worst injury” approach to generate estimates of years lived with disability (YLDs) [8], [9], with the assumption that the burden is accounted for by the first-reported injury. Other studies have focused only on multiply injured patients precluding comparison of isolated injury and multiple injury outcomes [4], [10]. Further studies have assessed the influence of injuries sustained in addition to a specific injury type on outcome, for example, whether polytraumatised traumatic brain injury (TBI) or spinal cord injury (SCI) patients experience poorer outcomes than patients with single injury diagnoses of TBI or SCI [11], [12], [13].

Improved understanding of how the number of injuries sustained relates to disability outcomes is needed to better characterise long term injury disability, inform the methodology of burden of injury studies, and reduce the potential for under-estimating the burden of injury. The aim of this study was to establish the association between the number of injuries sustained and disability outcomes 12-months post-injury for hospitalised patients.

## Methods

### Setting

This study is part of the Validating and Improving injury Burden Estimates Study (Injury-VIBES), which is described in detail elsewhere [14]. The primary aim of Injury-VIBES is to provide valid estimates of the burden of non-fatal injury using empirical data, through pooled analysis of de-identified, patient-level data from participants in six prospective cohort studies. The project was approved by the Monash University Human Research Ethics Committee for the provision and analysis of de-identified (anonymised) data from each participating cohort.

### Participants

Adult (18 years and over) cases from the Australian Victorian State Trauma Registry (VSTR) [15], [16] and Victorian Orthopaedic Trauma Outcomes Registry (VOTOR) [17], New Zealand Prospective Outcomes of Injury study (POIS) [18], and the USA National Study on Costs and Outcomes of Trauma (NSCOT) [19] were extracted for analysis. The remaining Injury-VIBES participating cohorts were not included here because the number of injuries recorded for each patient was capped, preventing full analysis of the number of injuries sustained. A summary of the data contributed by each source to the analysis of each disability outcome is provided in Table 1.

**Table 1:** Summary of participating datasets.

Dataset	Study timeframe	Inclusion Criteria	Number of participants	Percentage with multiple injuries	Outcome	Follow-up rate at 12-months
National Study on Costs and Outcomes of Trauma (USA)	Jul 2001–Nov 2002	18–85 years of age, ≥1 Abbreviated Injury Scale injury with a severity score >2	3920	GBD^a^ injury types 61%, ICD-10 body regions 71%	GOS-E^b^	83%
					SF-12^c^	77%
Victorian Orthopaedic Trauma Outcomes Registry (Australia)	Mar 2007–Mar 2011	Admitted to one of four hospitals with an orthopaedic injury >24 hours	15457	GBD injury types 42%, ICD-10 body regions 48%	GOS-E^a^	89%
		≥18 years of age			SF-12^b^	53%
					EQ-5D	62%
Victorian State Trauma Registry (Australia)	Jan 2007–Mar 2011	Injury Severity Score >15, ICU admission >24h, Urgent Surgery	7752	GBD injury types 76%, ICD-10 body regions 88%	GOS-E EQ-5D a	83% 55%
		≥18 years of age			SF-12^b^	52%
Prospective Outcome of Injury Study (NZ)	Dec 2007–Aug 2009	18–64 years, Accident Compensation Corporation entitlement claim, Admitted to hospital within 7 days of injury	711	GBD injury types 32%, ICD-10 body regions 38%	EQ-5D	80%

a GBD, Global Burden of Disease study;

b GOS-E, Glasgow Outcome Scale – Extended;

c SF-12, 12-item Short Form Health Survey.

### Definition of multiple injuries

Two approaches were used to define multiple injuries:

- *Number of 2010 Global Burden of Disease (GBD) study injury health states represented*
  International Classification of Disease 10^th^ Revision (ICD-10) diagnosis codes were mapped to the 23 injury types distinguished in health states of the 2010 GBD study [20]. Indicator variables were generated for the presence or absence of each 2010 GBD injury health state. The number of injury types represented was used to define the presence of a single or multiple injuries.
- *Number of ICD-10 body regions injured*
  ICD-10 diagnosis codes were mapped to variables indicating the presence or absence of an injury in each of the 12 ICD-10 body regions (head; neck; thorax; abdomen, lower back, lumbar spine and pelvis; shoulder and upper arm; elbow and forearm; wrist and hand; hip and thigh; knee and lower leg; ankle and foot; burns; all other Chapter 19 T-prefix injuries). The number of ICD-10 body regions represented was used to define the presence of a single or multiple injuries.

The NSCOT cases were mapped from the 9^th^ revision of the ICD (ICD-9-CM) codes to ICD-10 for consistency with the other datasets. For all datasets, the ICD diagnosis codes were obtained from the routine hospital discharge datasets of each relevant jurisdiction.

### Outcome measures

As all datasets followed up participants at 12-months post-injury, this time point was used for analysis. The methods used for follow-up are described in detail elsewhere for the VSTR and VOTOR [16], NSCOT [19] and POIS [18]. All studies collected outcomes at 12 months using standardised telephone interviews. The VSTR, VOTOR and NSCOT allowed interview by proxy where patients were unable to participate in the interview due to their physical or cognitive state. Three measures of outcome were used in this study:

- The Glasgow Outcome Scale – Extended (GOS-E) is used to measure patient function on a scale from 1 (death) to 8 (upper good recovery) [21]. While the GOS-E was developed for measuring head injury outcomes, the GOS-E is recommended for use in major trauma populations as it can be administered by proxy, includes most domains from the World Health Organization's International Classification of Functioning, Disability and Health [22], and is responsive to change in the non-head injured population [23]. The GOS-E was dichotomised for analysis with a score of 7 or higher representing a “good recovery” and a score less than 7 representing a “poor recovery”. A good recovery is where participants have returned to pre-injury levels of function with minimal or no injury-related sequelae. Dichotomization is the most widely used approach to analysis of the GOS-E [24]. Although it has been argued that analysing the GOS-E as an ordinal scale yields improved statistical efficiency over dichotomisation, the purpose of this study was to assess the presence or absence of disability, rather than the scale of disability, supporting dichotomisation of the GOS-E,
- The EQ-5D is a generic measure of health status which includes five items (mobility, self-care, usual activities, pain or discomfort, anxiety or depression) [25]. In a published consensus statement, the EQ-5D has been recommended for use in injury outcome studies [26] and has been widely used in injury studies [27]. Responses to each EQ-5D item were dichotomised into “no problems” and “some/severe problems” for analysis, an approach that has been widely used previously [28], and was consistent with the aim of this study to assess the presence or absence of disability.
- The 12-item Short Form Health Survey (SF-12) is a generic measure of health status which has component summary scores for mental (MCS-12) and physical (PCS-12) health [29].

### Data analysis

Categorical variables were summarised using counts and percentages; continuous variables with means and standard deviations (SD). Independent t-tests were used to compare groups where the variable was normally distributed and chi-square statistics were used for categorical variables. The numbers of 2010 GBD injury types, and ICD-10 body regions, represented were categorised for analysis (1, 2, 3, 4, 5, 6, 7 and 8+); patients with a single injury was the reference category. Age was categorised for analysis. For the GOS-E and EQ-5D items, the association between number of injuries and outcome was modelled using modified Poisson regression with a robust variance estimator [30]. Linear regression was used for the PCS-12 and MCS-12 outcomes. All models were adjusted for the age and sex of the patient, and the contributing source of data. Adjusted relative risks (ARR) and the corresponding 95% confidence intervals (CI) are presented for the modified Poisson models, and adjusted mean differences and 95% CI for the linear models. All analyses were performed using Stata Version 13 (StataCorp, College Station, TX, USA).

## Results

### Overview of participants

**Figure 1:**
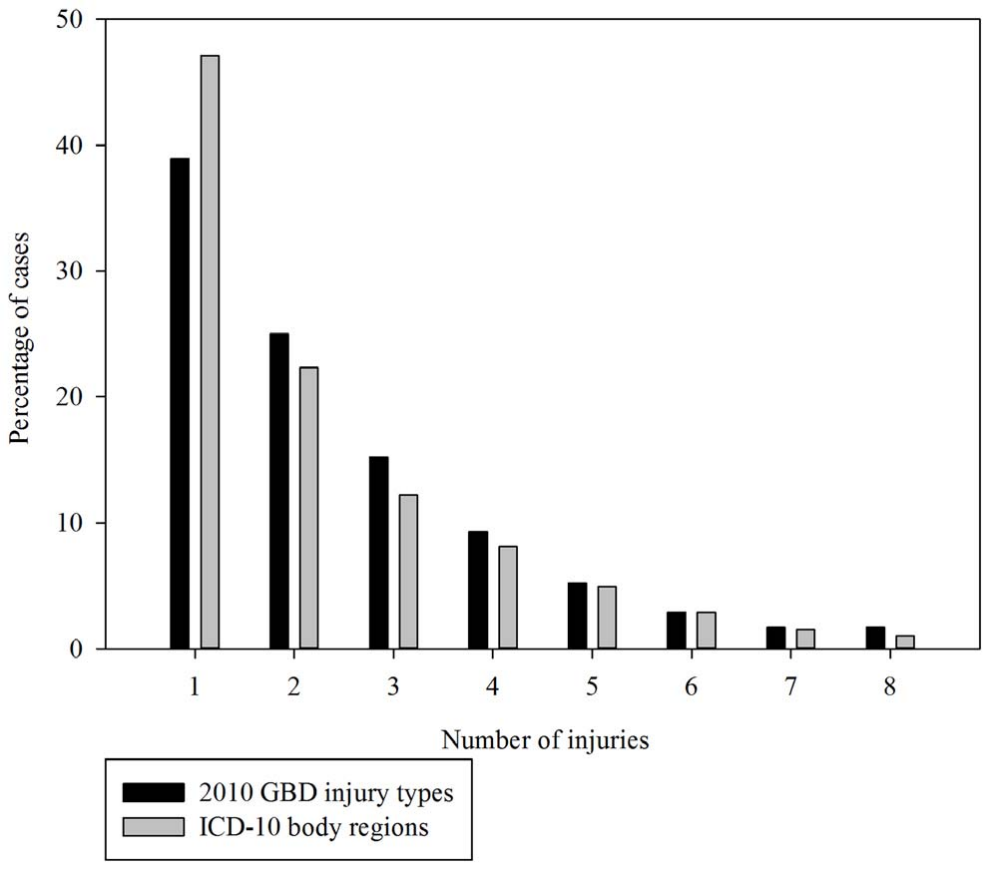
Proportion of cases by number of injuries sustained.

There were 27,840 eligible participants. The proportion of cases by number of injuries sustained using the two definitions of multiple injuries is shown in Fig. 1. Sixty-two percent (17,348) of the cases had sustained more than one GBD 2010 injury type, and 54% (n = 15,005) had sustained injuries to more than one ICD-10 body region. The profile of participants by multiple injury status is shown in Table 2. Age ranged from 18-110 years (mean: 52.8 years; SD: 22.6); and 59% were male. The mean age of multiply injured patients was younger than single injury cases, and a higher proportion was male and injured in transport-related events (Table 2). The GBD injury health types with the lowest prevalence of multiple injuries (i.e. the GBD types for which it was most often the case that only one injury diagnosis code was in the record) were dislocation of the hip, knee or shoulder, hip fracture, open wounds and superficial injuries, and fracture of the radius or ulna (S1 Table in S1 File). The GBD injury health types that most often had codes for additional injury types as well as the one recorded as the principal diagnosis (i.e. were most often multiple injury cases) were nerve injury, severe chest injury, spinal cord injury and fracture of the sternum, rib or face.

**Table 2:** Profile of participants according to multiple injury status.

Population descriptor		Number of GBD^a^ injury types			Number of ICD-10 body regions injured		
		Single injury	Multiple injuries	Test statistic	Single injury	Multiple injuries	Test statistic
		(n = 10,492)	(n = 17,348)	(p-value)	(n = 12,835)	(n = 15,005)	(p-value)
Age	Mean (SD) years	59.2 (22.7)	49.0 (21.7)	t = 37.5 (<0.001)	57.1 (22.8)	49.1 (21.8)	t = 29.9 (<0.001)
Sex	N (%)			Χ^2^_1_ = 1000 (<0.001)			Χ^2^_1_ = 621 (<0.001)
	Male	4942 (47.1)	11571 (66.7)		6597 (51.4)	9918 (66.1)	
	Female	5550 (52.9)	5777 (33.3)		6238 (48.6)	5087 (33.9)	
Cause of injury^b^	N (%)			Χ^2^_2_ = 3900 (<0.001)			Χ^2^_2_ = 4400 (<0.001)
	Falls	7234 (69.0)	6090 (35.1)		5836 (64.4)	5059 (33.7)	
	Transport	1318 (12.6)	8210 (47.4)		1793 (14.0)	7735 (51.6)	
	Other	1933 (18.4)	3038 (17.5)		2767 (21.6)	2204 (14.7)	

a GBD, Global Burden of Disease study;

b Data missing for n = 17 participants.

### Functional outcome – GOS-E good recovery (GOS-E>6)

Valid GOS-E scores at 12-months post-injury were recorded for 86.4% of participants. The follow-up rates were 83% for NSCOT, 83% for VSTR, and 89% for VOTOR cases. The proportion of multiply injured patients was similar for the patients followed up (53.8%) and those lost to follow-up (58.7%) (S2 Table in S2 File). The relative risks of poor recovery were significantly higher for all patients in the multiple injury categories, compared to the group sustaining a single injury, and increased as the number of injuries increased (Table 3). Treating the number of injuries as a continuous covariate, the relative risk of a poor functional recovery increased 8% (ARR 1.08, 95% CI: 1.07, 1.09) for each additional 2010 GBD injury type, and 7% (ARR 1.07, 95% CI: 1.06, 1.08) for each additional ICD-10 body region injured.

**Table 3:** Association between number of injuries sustained and a poor recovery (GOS-E<7) at 12-months.

Number of injuries	GBD^a^ injury types				ICD-10 body regions^c^ injured			
	N	N (%)	ARR^b^ (95% CI)	p-value	N	N (%)	ARR (95% CI)	p-value
		poor recovery				poor recovery		
1 (reference)	8907	4689 (52.6)	1.00		10829	5646 (52.1)	1.00	
2	5866	3114 (53.1)	1.06 (1.03, 1.10)	<0.001	5241	2865 (54.7)	1.08 (1.05, 1.11)	<0.001
3	3589	1924 (53.6)	1.12 (1.08, 1.16)	<0.001	2886	1624 (56.3)	1.14 (1.10, 1.19)	<0.001
4	2192	1258 (57.4)	1.25 (1.20, 1.31)	<0.001	1936	1109 (57.3)	1.21 (1.16, 1.27)	<0.001
5	1285	783 (60.9)	1.35 (1.28, 1.43)	<0.001	1194	739 (61.9)	1.28 (1.22, 1.35)	<0.001
6	736	496 (67.4)	1.49 (1.40, 1.58)	<0.001	736	508 (69.0)	1.44 (1.36, 1.52)	<0.001
7	422	316 (74.9)	1.64 (1.54, 1.74)	<0.001	368	266 (72.3)	1.48 (1.38, 1.59)	<0.001
8+	446	377 (84.5)	1.83 (1.73, 1.92)	<0.001	253	200 (79.1)	1.57 (1.47, 1.68)	<0.001

a GBD, Global Burden of Disease study;

b ARR, adjusted relative risk compared to the reference group (1 injury)– adjusted for age, gender and data source (study);

c ICD-10 body regions: head; neck; thorax; abdomen, lower back, lumbar spine and pelvis; shoulder and upper arm; elbow and forearm; wrist and hand; hip and thigh; knee and lower leg; ankle and foot; burns; all other T-prefix injuries.

### EQ-5D

Valid EQ-5D responses were recorded for 60.1% of participants. The follow-up rates were 55% for VSTR, 62% for VOTOR and 80% for POIS, reflecting the late inclusion of the EQ-5D to the VSTR and VOTOR follow-up protocols. The proportion of multiply injured patients was similar for the patients followed up (51.6%) and those lost to follow-up (54.6%) (S2 Table in S1 File). The relative risk of reporting problems on the usual activities and anxiety/depression items at 12-months was significantly higher for patients in the multiple injury categories, compared to the group sustaining a single injury, and increased as the number of injuries sustained increased (Table 4). This pattern was also noted for the mobility item of the EQ-5D when multiple injuries was defined using the number of ICD-10 body regions injured, and for the anxiety/depression item when considering multiple GBD 2010 injury types (Table 4). For both definitions of multiple injury, the relative risks of reporting problems with self-care were only significantly higher for cases sustaining six or more injuries, when compared to the single injury group (Table 4).

**Table 4:** Association between number of injuries sustained and reporting limitations on each EQ-5D item at 12-months.

EQ-5D Item	Number of injuries	GBD^a^ injury types				ICD-10 body regions^c^ injured			
		N	N (%)	ARR^b^ (95% CI)	p-value	N	N (%)	ARR (95% CI)	p-value
			With problems				With problems		
**Mobility**	1 (reference)	5845	2871 (49.1)	1.00		6956	3255 (46.8)	1.00	
	2	3566	1592 (44.6)	1.02 (0.98, 1.06)	0.42	3156	1498 (47.5)	1.08 (1.04, 1.12)	<0.001
	3	2068	865 (41.8)	1.09 (1.03, 1.14)	0.002	1630	696 (42.7)	1.08 (1.02, 1.14)	0.01
	4	1245	519 (41.7)	1.24 (1.16, 1.33)	<0.001	1088	442 (40.6)	1.17 (1.09, 1.26)	<0.001
	5	708	279 (39.4)	1.25 (1.13, 1.38)	<0.001	708	310 (43.8)	1.27 (1.16, 1.39)	<0.001
	6	412	196 (47.6)	1.55 (1.39, 1.73)	<0.001	421	213 (50.6)	1.58 (1.42, 1.76)	<0.001
	7	251	132 (52.6)	1.77 (1.56, 2.01)	<0.001	241	128 (53.1)	1.70 (1.50, 1.93)	<0.001
	8+	272	196 (72.1)	2.59 (2.35, 2.85)	<0.001	167	108 (64.7)	2.02 (1.78, 2.30)	<0.001
**Self-care**	1 (reference)	5835	1864 (32.0)	1.00		6944	2079 (29.9)	1.00	
	2	3559	943 (26.5)	0.95 (0.90, 1.00)	0.07	3150	886 (28.1)	1.01 (0.95, 1.07)	0.73
	3	2067	480 (23.2)	0.96 (0.89, 1.04)	0.37	1628	392 (24.1)	0.99 (0.91, 1.07)	0.78
	4	1242	255 (20.5)	1.03 (0.92, 1.15)	0.62	1089	225 (20.7)	1.03 (0.92, 1.16)	0.60
	5	709	143 (20.2)	1.10 (0.93, 1.29)	0.27	708	151 (21.3)	1.06 (0.92, 1.16)	0.45
	6	412	104 (25.2)	1.39 (1.16, 1.66)	<0.001	421	109 (25.9)	1.44 (1.21, 1.71)	<0.001
	7	252	68 (27.0)	1.56 (1.27, 1.92)	<0.001	242	64 (26.5)	1.53 (1.23, 1.91)	<0.001
	8+	273	105 (38.5)	2.48 (2.08, 2.96)	<0.001	167	56 (33.5)	1.85 (1.46, 2.35)	<0.001
**Usual activities**	1 (reference)	5834	3092 (53.0)	1.00		6944	3628 (52.3)	1.00	
	2	3561	1916 (53.8)	1.09 (1.05, 1.13)	<0.001	3151	1751 (55.6)	1.10 (1.06, 1.14)	<0.001
	3	2060	1111 (53.9)	1.17 (1.12, 1.23)	<0.001	1623	909 (56.0)	1.17 (1.11, 1.23)	<0.001
	4	1243	689 (55.4)	1.30 (1.23, 1.38)	<0.001	1087	596 (54.8)	1.23 (1.16, 1.30)	<0.001
	5	707	400 (56.6)	1.36 (1.27, 1.47)	<0.001	708	414 (58.5)	1.31 (1.22, 1.41)	<0.001
	6	412	256 (62.1)	1.52 (1.39, 1.65)	<0.001	420	266 (63.3)	1.46 (1.35, 1.58)	<0.001
	7	252	171 (67.9)	1.68 (1.53, 1.85)	<0.001	242	153 (63.2)	1.48 (1.33, 1.63)	<0.001
	8+	273	211 (77.3)	1.97 (1.82, 2.13)	<0.001	167	129 (77.3)	1.76 (1.61, 1.93)	<0.001
**Pain/discomfort**	1 (reference)	5783	2895 (50.1)	1.00		6886	3410 (49.5)	1.00	
	2	3537	1886 (53.3)	1.11 (1.06, 1.15)	<0.001	3126	1738 (55.6)	1.15 (1.10, 1.19)	<0.001
	3	2053	1145 (55.7)	1.22 (1.16, 1.28)	<0.001	1620	924 (57.0)	1.21 (1.15, 1.27)	<0.001
	4	1235	711 (57.6)	1.32 (1.24, 1.40)	<0.001	1084	648 (59.8)	1.31 (1.24, 1.39)	<0.001
	5	710	421 (60.0)	1.40 (1.31, 1.51)	<0.001	702	425 (60.5)	1.34 (1.26, 1.44)	<0.001
	6	412	264 (64.4)	1.53 (1.41, 1.66)	<0.001	419	282 (67.3)	1.52 (1.41, 1.63)	<0.001
	7	250	183 (72.9)	1.76 (1.61, 1.92)	<0.001	238	162 (68.1)	1.54 (1.40, 1.69)	<0.001
	8+	271	207 (76.7)	1.86 (1.71, 2.02)	<0.001	166	123 (74.1)	1.66 (1.50, 1.83)	<0.001
**Anxiety/depression**	1 (reference)	5755	1994 (34.7)	1.00		6855	2411 (35.2)	1.00	
	2	3529	1338 (37.9)	1.12 (1.06, 1.18)	<0.001	3112	1244 (40.0)	1.14 (1.08, 1.20)	<0.001
	3	2041	864 (42.3)	1.27 (1.19, 1.35)	<0.001	1614	696 (43.1)	1.22 (1.15, 1.31)	<0.001
	4	1229	551 (44.8)	1.37 (1.26, 1.48)	<0.001	1081	458 (42.4)	1.22 (1.13, 1.33)	<0.001
	5	703	339 (48.2)	1.46 (1.33, 1.61)	<0.001	703	340 (48.4)	1.38 (1.27, 1.51)	<0.001
	6	410	211 (51.5)	1.54 (1.38, 1.72)	<0.001	417	221 (53.0)	1.50 (1.36, 1.66)	<0.001
	7	251	127 (50.6)	1.52 (1.32, 1.73)	<0.001	238	111 (46.6)	1.32 (1.14, 1.53)	<0.001
	8+	268	150 (56.0)	1.67 (1.48, 1.89)	<0.001	166	93 (56.0)	1.53 (1.33, 1.77)	<0.001

a GBD, Global Burden of Disease study;

b ARR, adjusted relative risk compared to the reference group (1 injury) – adjusted for age, gender and data source (study);

c ICD-10 body regions: head; neck; thorax; abdomen, lower back, lumbar spine and pelvis; shoulder and upper arm; elbow and forearm; wrist and hand; hip and thigh; knee and lower leg; ankle and foot; burns; all other T-prefix injuries.

For each additional 2010 GBD injury type, the adjusted relative risk of reporting problems on the EQ-5D items increased by 10% (ARR 1.10, 95% CI: 1.09, 1.12) for mobility, 8% (ARR 1.08, 95% CI: 1.06, 1.10) for self-care, 9% (ARR 1.09, 95% CI: 1.08, 1.10) for usual activities, 9% (ARR 1.09, 95% CI: 1.08, 1.10) for pain/discomfort, and 8% (ARR 1.08, 95% CI: 1.07, 1.10) for anxiety/depression. Similarly, for each additional ICD-10 body region injured, the adjusted relative risk of reporting problems on the EQ-5D items increased by 8% (ARR 1.08, 95% CI: 1.07, 1.10) for mobility, 5% (ARR 1.05, 95% CI: 1.03, 1.07) for self-care, 7% (ARR 1.07, 95% CI: 1.06, 1.08) for usual activities, 8% (ARR 1.08, 95% CI: 1.07, 1.09) for pain/discomfort, and 7% (ARR 1.07, 95% CI: 1.06, 1.08) for anxiety/depression.

### SF-12

**Figure 2:**
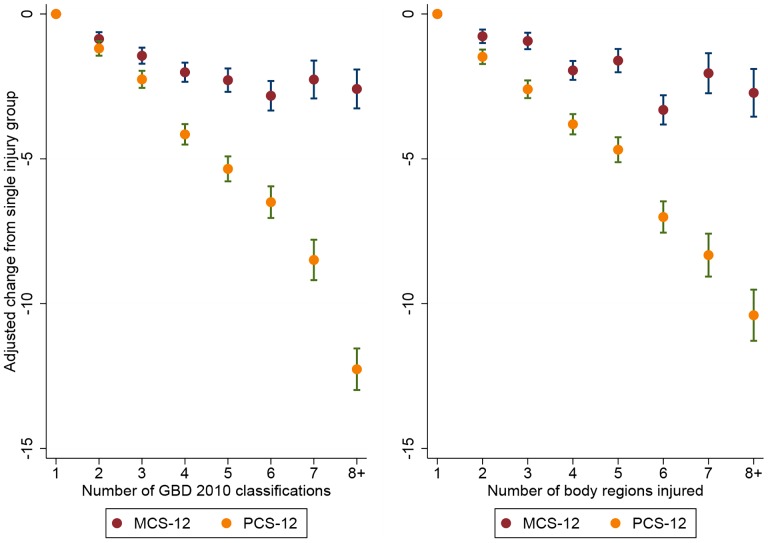
Adjusted difference in mean MCS-12 and PCS-12 scores by number of injuries sustained (adjusted for age, gender and data source (study)).

Valid PCS-12 and MCS-12 scores were collected for 56.4% of participants. The proportion of multiply injured patients was similar for the patients followed-up (57.1%) and those lost to follow-up (51.1%) (S2 Table in S1 File). The adjusted mean PCS-12 score declined significantly as the number of injuries sustained increased (Fig. 2). While the adjusted mean MCS-12 score for each of the multiple injury categories was lower than the single injury group, the degree of decline largely plateaued after more than three injuries (Fig. 2). For each additional 2010 GBD injury type or ICD-10 body region injured, the adjusted mean PCS-12 score decreased 1.5 (95% CI: 1.3, 1.6) or 1.3 (95% CI: 1.2, 1.5) points, respectively. The adjusted mean MCS-12 score decreased by 0.5 (95% CI: 0.4, 0.6) points for each additional injury sustained, regardless of definition used.

## Discussion

Despite the potential for multiple injuries to lead to increased disability after injury, there is limited published evidence of the association between the number of injuries sustained and longer term functional and quality of life outcomes. We found a strong association between the number of GBD injury types, and ICD-10 body regions injured, and disability outcomes at 12-months. There was a large difference in the proportion of patients experiencing a poorer outcome when comparing single injury cases to the multiply injured, particularly for measures of physical functioning and pain. The difference between cases with eight or more types of injuries, or body regions injured, and cases with a single injury averaged more than 20% for the GOS-E (27–32%) and for the EQ-5D mobility (18–23%), usual activities (22–24%), and pain/discomfort (19–27%) outcomes.

A challenge for this study was defining multiple injuries in the absence of an agreed definition in the literature. One definition of multiple injuries, or polytrauma, is when injuries occur simultaneously in multiple parts of the body [31], while others consider injuries to two or more simultaneous diagnostic groups as multiple injuries [1]. We used both approaches separately, and the findings were consistent regardless of whether ‘multiple injuries’ was defined as the number of ICD-10 body regions with an injury or the number of 2010 GBD injury types represented.

In previous injury burden studies, including the 2010 GBD study, only the first injury was considered when calculating non-fatal burden, with the underlying assumption that additional injuries do not contribute further burden warranting measurement. In our study of hospitalised injury cases, multiple injuries were common; a finding consistent with Aharonson-Daniel et al who reported that 52% of hospitalised trauma patients in Israel had more than one diagnosis and 39% had a diagnosis in more than one body region [1]. For most outcomes in our study, the relative risk of poorer outcomes at 12-months post-injury was higher for all multiple injury categories, suggesting that even one additional injury has important implications for disability outcomes.

Previous studies have predominantly relied on the ISS or NISS to characterise multiple injuries for predicting disability outcomes [2], [3], [4], [5], [6], with conflicting findings^3–6^. As the ISS uses a “threat to life scale” and includes a maximum of three injuries, it does not provide a comprehensive representation of the injury profile. Injuries that could represent a high risk of disability (e.g. fractures) may not contribute to the ISS/NISS calculation if three or more life-threatening injuries are present. Yet once the threat of death has passed, some injuries contributing to the ISS or NISS (e.g. ruptured spleen) may result in little subsequent disability compared to injuries such as a fracture. Meerding et al found that the number of injuries sustained was a significant predictor of functioning up to 9-months post-injury, but like the ISS, only a maximum of three injuries were considered in their study [7]. One in five hospitalised injury patients in our study had sustained more than three injuries, and as the number of injuries increased, the risk of a poorer outcome also increased.

Few studies have assessed the relationship between the presence of multiple injuries and a range of functional or quality of life outcomes. Holtslag et al studied 359 major trauma patients and failed to find a significant association between the ISS and the GOS or with each of the EQ-5D items 12–18 months post-injury [3]. Similarly, in a study of 105 patients with a NISS >15, Soberg et al found that the NISS was not a predictor of any of the sub-scales of the SF-36 at one or two years post-injury [32]. In contrast, Ringburg et al studied 246 severely injured trauma patients and found that the ISS was a significant predictor of EQ-5D mobility, usual activities and self-care items, but was not an important predictor of anxiety/depression or pain/discomfort 12-months after injury [5]. We found a strong dose-response relationship, between the number of injury types, and body regions injured, and pain and the more “physical” measures of disability such as a GOS-E good recovery, EQ-5D mobility and usual activities, and the PCS-12, a finding mostly consistent with Ringburg et al. Notably, there was a similar but less pronounced pattern of increased risk of poorer outcome with the presence of multiple injuries for the EQ-5D anxiety/depression item and the MCS-12 (Table 4 and Fig. 2).

The study strengths were the very large study sample, relative heterogeneity in the spectrum of injury, and multiple measures of disability at a consistent time point post-injury. Using data from injured persons from several countries, health jurisdictions, and datasets was both a strength and a limitation. The inclusion criteria of the datasets differed, ranging from any admission to hospital or treatment for greater than 3 hours in the emergency department (POIS), to an in-hospital stay of at least 24 hours (VOTOR) to serious injuries only based on ISS and other criteria (VSTR and NSCOT), and this is reflected in the proportion of cases with multiple injuries across the datasets. Data from the contributing studies were also collected over different calendar years. Acknowledging this variability, the analysis for this study was limited to adult patients only who were hospitalised due to injury, and all models were adjusted for data source to ensure estimates were independent of inherent differences in time and setting. The overall pattern of the association between the number of injuries and outcomes was consistent for each dataset when analysed separately, though the precision of the estimates was lower due to the smaller number of cases in each individual study (data tables available on request).

Follow-up rates were consistently high for the GOS-E outcome across all studies, as this instrument can be administered reliably by proxy, but there was greater loss to follow-up for the EQ-5D and the SF-12 (Table 1). The late inclusion of the EQ-5D in the VSTR and VOTOR study protocols also reduced the number of cases available for analysis of this outcome, though should introduce no additional bias to the study as the follow-up rates for this instrument since inclusion were comparable to the POIS. The lower SF-12 completion rates are explained partly by the high prevalence of serious traumatic brain injury in the NSCOT and VSTR cases, while both the VSTR and VOTOR completion rates were impacted by the inclusion of a higher proportion of cases in older adults where direct interviews with patients can be challenging due to higher prevalence of pre-existing cognitive deficits and general frailty. In addition, the VSTR and VOTOR include all cases and do not exclude patients with characteristics such as extreme age, frailty, cognitive deficit, inability to communicate in English, or the lack of a fixed address. The SF-12 cannot be administered validly to many such cases. Therefore, while the wide inclusion criteria of VSTR and VOTOR contributed to relatively low SF-12 follow-up and could have introduced responder bias, the cases that were followed up are predominantly those where the SF-12 can be administered validly. Previous studies have shown higher loss to follow-up in less severely injured patients but the reasons for loss to follow-up, and whether they are related to better or poorer outcomes, have not been clearly established [7], [33], [34]. Only the 12-month follow-up data are presented in this paper, but the findings were consistent at 6 and 24-months post-injury. Six-month data were available for POIS, VOTOR and the VSTR studies, and 24-month data were available for the POIS and VSTR datasets (S2–S6 Tables in S1 File).

It was beyond the scope of this paper to establish the level of disability associated with particular patterns of injuries and this remains an area for further investigation. Finally, the definitions of multiple injuries used in our study excluded multiple diagnoses within a single ICD-10 body region or GBD injury type (e.g. bilateral fractures, etc.). Further investigation of the impact of multiple diagnoses within a single body region on disability outcomes is needed.

Overall, in this study of more than 20,000 injured participants, there was a consistent and strong relationship between the number of injuries sustained and 12-month functional and health status outcomes. Each additional injury type or body region injured increased the risk of a poor functional outcome by 5–10%. Existing composite measures of anatomical injury severity such as the NISS or ISS may be insufficient to characterise and account for multiple injuries in disability studies. Future studies should consider the impact of multiple injuries to avoid under-estimation of injury burden. As the use of functional and quality of life measures increases in routine practice, studies will need to take multiple injuries into account in any analyses comparing system or centre performance.

## Supporting Information

S1 File S1–S6 Tables. **S1 Table.** Number and percentage of GBD 2010 injury types represented according for each principal diagnosis injury health state. **S2 Table.** Comparison of cases lost to follow-up and cases successfully followed up at 12 months post-injury. **S3 Table.** Association between number of ICD-10 body regions injured and 6-month disability outcomes. **S4 Table.** Association between number of 2010 GBD injury types represented and 6-month disability outcomes. **S5 Table.** Association between number of ICD-10 body regions injured and 24-month disability outcomes. **S6 Table.** Association between number of 2010 GBD injury types represented and 24-month disability outcomes.
(DOCX)

## References

[1] Aharonson-Daniel L, Boyko V, Ziv A, Avitzour M, Peleg K (2003) A new approach to the analysis of multiple injuries using data from a national trauma registry. Inj Prev 9:156–162. 12810744 10.1136/ip.9.2.156 PMC1730962

[2] Holtslag H, Post M, Lindeman E, Van der Werken C (2007) Long-term functional health status of severely injured patients. Injury 38:280–289. 17250834 10.1016/j.injury.2006.10.026

[3] Holtslag H, Van Beeck E, Lindeman E, Leenen L (2007) Determinants of long-term functional consequences after major trauma. J Trauma 62:919–927. 17426549 10.1097/01.ta.0000224124.47646.62

[4] Pape H, Probst C, Lohse R, Zelle B, Panzica M, et al (2010) Predictors of late clinical outcome following orthopedic injuries after multiple trauma. J Trauma 69:1243–1251. 20489671 10.1097/TA.0b013e3181ce1fa1

[5] Ringburg A, Polinder S, van Ierland M, Steyerberg E, van Lieshout E, et al (2011) Prevalence and prognostic factors of disability after major trauma. J Trauma 70:916–922. 21045741 10.1097/TA.0b013e3181f6bce8

[6] Soberg H, Finset A, Bautz-Holter E, Sandvik L, Roise O (2007) Return to work after severe multiple injuries: A multidimensional approach on Status 1 and 2 years postinjury. J Trauma 62:471–481. 17297338 10.1097/TA.0b013e31802e95f4

[7] Meerding W, Looman C, Essink-Bot M, Toet H, Mulder S, et al (2004) Distribution and determinants of health and work status in a comprehensive population of injury patients. J Trauma 56:150–161. 14749582 10.1097/01.TA.0000062969.65847.8B

[8] Mathers C, Vos E, Stevenson C, Begg S (2001) The burden of disease and injury in Australia. Bull World Health Org 79:1076–1084. 11731817 PMC2566696

[9] Vos T, Flaxman A, Naghavi M (2012) Years lived with disability (YLDs) for 1160 sequelae of 289 diseases and injuries 1990–2010: a systematic analysis for the Global Burden of Disease Study 2010. Lancet 380:2163–2196. 23245607 10.1016/S0140-6736(12)61729-2 PMC6350784

[10] Weninger P, Aldrian S, Koenig F, Vecsei V, Nau T (2008) Functional recovery at a minimum of 2 years after multiple injury - Development of an outcome score. J Trauma 65:799–808. 18849794 10.1097/TA.0b013e3181820dae

[11] Putz C, Schuld C, Gantz S, Grieser T, Akbar M, et al (2011) The effect of polytrauma as a possible confounder in the outcome of monotraumatic vs polytraumatic paraplegic patients: a clinical cohort study. Spinal Cord 49:721–727. 21243001 10.1038/sc.2010.181

[12] Scivoletto G, Farchi S, Laurenza L, Tamburella F, Molinari M (2013) Impact of multiple injuries on functional and neurological outcomes of patients with spinal cord injury. Scand J Trauma, Resusc Emerg Med 21:42. 23718823 10.1186/1757-7241-21-42 PMC3669625

[13] Gross T, Schuepp M, Attenberger C, Pargger H, Amsler F (2012) Outcome in polytraumatized patients with and without brain injury. Acta Anaesth Scand 56:1163–1174. 22735047 10.1111/j.1399-6576.2012.02724.x

[14] Gabbe B, Lyons R, Harrison J, Rivara F, Ameratunga S, et al. (2013) Validating and Improving Injury Burden Estimates Study: the Injury-VIBES study protocol. Inj Prev. doi:10.1136/injuryprev-2013-040936. 10.1136/injuryprev-2013-040936 23920023

[15] Cameron P, Finch C, Gabbe B, Collins L, Smith K, et al (2004) Developing Australia's first statewide trauma registry - What are the lessons? Aust N Z J Surg 74:424–428. 10.1111/j.1445-1433.2004.03029.x 15191472

[16] Gabbe B, Sutherland A, Hart M, Cameron P (2010) Population-based capture of long-term functional and quality of life outcomes after major trauma - the experiences of the Victorian State Trauma Registry. J Trauma 69:532–536. 20838122 10.1097/TA.0b013e3181e5125b

[17] Edwards E, Graves S, McNeil J, Williamson O, Urquhart D, et al (2006) Orthopaedic trauma: Establishment of an outcomes registry to evaluate and monitor treatment effectiveness. Injury 37:95–96. 15979074 10.1016/j.injury.2005.02.027

[18] Derrett S, Langley J, Hokowhitu B, Ameratunga S, Hansen P, et al (2009) Prospective outcomes of injury study. Inj Prev 15 doi:10.1136/ip.2009.022558 10.1136/ip.2009.022558a 19805606

[19] MacKenzie E, Rivara F, Jurkovich G, Nathens A, Frey K, et al (2007) The national study on costs and outcomes of trauma. J Trauma 63:S54–S67. 18091213 10.1097/TA.0b013e31815acb09

[20] Murray C, Vos T, Lozano R (2012) Disability-adjusted life years (DALYs) for 291 diseases and injuries in 21 regions, 1990–2010: a systematic analysis for the Global Burden of Disease Study 2010. Lancet 380:2197–2223. 23245608 10.1016/S0140-6736(12)61689-4

[21] Wilson J, Pettigrew L, Teasdale G (1998) Structured Interviews for the Glasgow Outcome Scale and the Extended Glasgow Outcome Scale: Guidlines for Their Use. J Neurotrauma 15:573–585. 9726257 10.1089/neu.1998.15.573

[22] Ardolino A, Sleat G, Willett K (2012) Outcome measurements in major trauma - Results of a consensus meeting. Injury 43:1662–1666. 22695320 10.1016/j.injury.2012.05.008

[23] Williamson O, Gabbe B, Forbes A, Wolfe R, Sutherland A, et al (2011) Comparing the responsiveness of functional outcome assessment instruments for trauma registries. J Trauma 71:63–68. 21427612 10.1097/TA.0b013e31820e898d

[24] Maas A, Steyerberg E, Marmarou A, McHugh G, Lingsma H, et al (2010) IMPACT recommendations for improving the design and analysis of clinical trials in moderate to severe traumatic brain injury. Neurotherapeutics 7:127–134. 20129504 10.1016/j.nurt.2009.10.020 PMC5084119

[25] Dolan P (1997) Modeling valuations for EuroQol health states. Med Care 35:1095–1108. 9366889 10.1097/00005650-199711000-00002

[26] Van Beeck E, Larsen C, Lyons R, Meerding W, Mulder S, et al (2007) Guidelines for the conduction of follow-up studies measuring injury-related disability. J Trauma 62:534–550. 17297349 10.1097/TA.0b013e31802e70c7

[27] Derrett S, Black J, Herbison G (2009) Outcome After Injury—A Systematic Literature Search of Studies Using the EQ-5D. J Trauma 67:883–890. 19820601 10.1097/TA.0b013e3181ae6409

[28] Black J, Herbison G, Lyons R, Polinder S, Derrett S (2011) Recovery after injury: an individual patient data meta-analysis of general health status using the EQ-5D. J Trauma 71:1003–1010. 21986741 10.1097/TA.0b013e3182238833

[29] Ware J, Kosinski M, Keller S (1998) SF-12: How to score the SF-12 physical and mental health summary scales. Lincoln, Rhode Island: QualityMetric Incorporated.

[30] Zou G (2004) A Modified Poisson Regression Approach to Prospective Studies with Binary Data. Am J Epidemiol 159:702–706. 15033648 10.1093/aje/kwh090

[31] Bouillon B, Kreder H, Eypasch E, Holbrook TL, Kreder HJ, et al (2002) Quality of life in patients with multiple injuries - basic issues, assessment, and recommendations. Restor Neurol Neurosci 20:125–134. 12454361

[32] Soberg H, Bautz-Holter E, Roise O, Finset A (2007) Long-Term Multidimensional Functional Consequences of Severe Multiple Injuries Two Years After Trauma: A Prospective Longitudinal Cohort Study. J Trauma 62:461–470. 17297337 10.1097/01.ta.0000222916.30253.ea

[33] Macpherson A, Rothman L, McKeag A, Howard A (2003) Mechanism of injury affects 6-month functional outcome in children hospitalized because of severe injuries. J Trauma 55:454–458. 14501886 10.1097/01.TA.0000042158.79688.51

[34] O'Donnell M, Holmes A, Creamer M, Ellen S, Judson R, et al (2009) The role of post-traumatic stress disorder and depressioin in predicting disability after injury. Med J Aust 190:S71–S74. 19351297 10.5694/j.1326-5377.2009.tb02474.x

