# Supplementary material for: Association between the Number of Injuries Sustained and 12-Month Disability Outcomes: Evidence from the Injury-VIBES Study
Source: PLoS One. 2014 Dec 11;9(12):e113467. doi: 10.1371/journal.pone.0113467 (PMC4263479; doi:10.1371/journal.pone.0113467)
Supplement: S1 File — S1–S6 Tables. S1 Table. Number and percentage of GBD 2010 injury types represented according for each principal diagnosis injury health state. S2 Table. Comparison of cases lost to follow-up and cases successfully followed up at 12 months post-injury. S3 Table. Association between number of ICD-10 body regions injured and 6-month disability outcomes. S4 Table. Association between number of 2010 GBD injury types represented and 6-month disability outcomes. S5 Table. Association between number of ICD-10 body regions injured and 24-month disability outcomes. S6 Table. Association between number of 2010 GBD injury types represented and 24-month disability outcomes. (DOCX) [file pone.0113467.s001.docx]

**Table S1: Number and percentage of GBD 2010 injury types represented according for each principal diagnosis injury health state**

| 2010 GBD Injury type principal diagnosis | Single injury | 2 injuries | 3 injuries | 4 injuries | 5 injuries | 6 injuries | 7 injuries | 8+ injuries |
| --- | --- | --- | --- | --- | --- | --- | --- | --- |
|  | **N (%)** | **N (%)** | **N (%)** | **N (%)** | **N (%)** | **N (%)** | **N (%)** | **N (%)** |
| Dislocation hip/knee/shoulder | 179 (69.4) | 56 (22.5) | 13 (5.0) | 5 [1.9] | 1 (0.4) | 2 (0.8) | 0 (0.0) | 0 (0.0) |
| Fracture of hip | 2283 (67.8) | 685 (20.3) | 200 (5.9) | 66 (2.0) | 31 (0.9) | 34 (1.0) | 25 (0.7) | 45 (1.4) |
| Open wound, superficial injuries & dislocations | 572 (60.3) | 237 (25.0) | 80 (8.4) | 45 (4.7) | 9 (1.0) | 6 (0.6) | 0 (0.0) | 0 (0.0) |
| Fracture of radius or ulna | 1610 (57.3) | 683 (24.3) | 311 (11.1) | 139 (5.0) | 42 (1.5) | 11 (0.4) | 9 (0.3) | 3 (0.1) |
| Burn <20% total body surface area | 57 (53.8) | 21 (19.8) | 16 (15.1) | 7 (6.6) | 3 (2.8) | 1 (0.9) | 1 (0.9) | 0 (0.0) |
| Fracture of patella, tibia, fibula or ankle | 1789 (47.0) | 1206 (31.7) | 466 (12.3) | 222 (5.8) | 74 (2.0) | 29 (0.8) | 12 (0.3) | 5 (0.1) |
| Fracture of pelvis | 557 (41.6) | 360 (26.9) | 209 (15.6) | 108 (8.1) | 77 (5.8) | 18 (1.3) | 6 (0.5) | 3 (0.2) |
| Fracture of clavicle, scapula, humerus or skull | 716 (40.4) | 555 (31.3) | 321 (18.1) | 114 (6.4) | 55 (3.1) | 10 (0.5) | 1 (0.1) | 1 (0.1) |
| Fracture of wrist or distal hand, fracture of foot | 299 (39.8) | 300 (40.0) | 99 (13.2) | 34 (4.5) | 14 (1.9) | 3 (0.4) | 1 (0.1) | 1 (0.1) |
| Amputation of finger | 16 (39.0) | 7 (17.1) | 6 (14.6) | 8 (19.5) | 2 (4.9) | 1 (2.4) | 0 (0.0) | 1 (2.4) |
| Fracture of femur | 443 (35.4) | 259 (20.7) | 153 (12.2) | 126 (10.1) | 93 (7.4) | 68 (5.4) | 53 (4.2) | 57 (4.6) |
| Fracture of vertebral column | 660 (34.2) | 648 (33.6) | 388 (20.1) | 160 (8.3) | 54 (2.8) | 16 (0.8) | 4 (0.2) | 0 (0.0) |
| Amputation of lower or upper extremity | 6 (23.1) | 7 (26.9) | 7 (26.9) | 2 (7.7) | 1 (3.9) | 1 (3.9) | 1 (3.9) | 1 (3.9) |
| Injury requiring emergency care | 187 (19.6) | 292 (30.7) | 209 (21.9) | 116 (12.2) | 77 (8.1) | 45 (4.7) | 11 (1.2) | 15 (1.6) |
| Severe traumatic brain injury | 829 (19.3) | 989 (23.0) | 858 (20.0) | 635 (14.8) | 365 (8.5) | 252 (5.9) | 168 (3.9) | 200 (4.7) |
| Fracture of sternum, rib or face bone | 25 (11.8) | 69 (32.5) | 71 (33.5) | 32 (15.1) | 10 (4.7) | 4 (1.9) | 1 (0.5) | 0 (0.0) |
| Spinal cord lesion at neck level | 36 (11.7) | 62 (20.2) | 66 (21.5) | 57 (18.6) | 36 (11.7) | 15 (4.9) | 13 (4.2) | 22 (7.2) |
| Spinal cord lesion below neck level | 19 (8.3) | 38 (16.6) | 43 (18.8) | 31 (13.5) | 35 (15.3) | 27 (11.8) | 9 (3.9) | 27 (11.8) |
| Other injury | 8 (8.1) | 26 (26.3) | 21 (21.2) | 17 (17.2) | 17 (17.2) | 7 (7.1) | 1 (1.0) | 2 (2.0) |
| Severe chest injury | 193 (6.4) | 516 (17.0) | 685 (22.6) | 655 (21.6) | 456 (15.0) | 268 (8.8) | 158 (5.2) | 103 (3.4) |
| Injury requiring urgent care | 10 (4.7) | 50 (23.7) | 57 (27.0) | 41 (19.4) | 21 (10.0) | 16 (7.6) | 9 (4.3) | 7 (3.3) |
| Burn ≥ 20% total body surface area | 0 (0.0) | 20 (27.8) | 29 (40.3) | 15 (20.8) | 7 (9.7) | 0 (0.0) | 0 (0.0) | 1 (1.4) |
| Injured nerves | 0 (0.0) | 9 (37.5) | 12 (50.0) | 0 (0.0) | 3 (12.5) | 0 (0.0) | 0 (0.0) | 0 (0.0) |

**Table S2: Comparison of cases lost to follow-up and cases successfully followed up at 12 months post-injury**

|  | **Followed up** | **Lost to follow-up** |
| --- | --- | --- |
|  | **n (%)** | **n (%)** |
| **Number of ICD-10 body regions** |  |  |
| **EQ-5D** | **(n=14367)** | **(n=9553)** |
| 1 | 6975 (48.5) | 4342 (45.4) |
| 2 | 3141 (21.9) | 2134 (22.3) |
| 3 | 1627 (11.3) | 1230 (12.9) |
| 4 | 1087 (7.6) | 782 (8.2) |
| 5 | 708 (4.9) | 498 (5.2) |
| 6 | 421 (2.9) | 314 (3.3) |
| 7 | 241 (1.7) | 149 (1.6) |
| 8+ | 167 (1.2) | 104 (1.1) |
| **GOS-E** | **(n=23443** | **(n=3686)** |
| 1 | 10829 (46.2) | 1522 (41.3) |
| 2 | 5241 (22.3) | 913 (24.8) |
| 3 | 2886 (12.3) | 531 (14.4) |
| 4 | 1936 (8.3) | 347 (9.4) |
| 5 | 1194 (5.1) | 194 (5.2) |
| 6 | 736 (3.1) | 98 (2.7) |
| 7 | 368 (1.6) | 51 (1.4) |
| 8+ | 253 (1.1) | 30 (0.8) |
| **SF-12** | N = 15311 | N = 11818 |
| 1 | 6567 (42.9) | 5784 (48.9) |
| 2 | 3404 (22.2) | 2750 (23.3) |
| 3 | 2006 (13.1) | 1411 (11.9) |
| 4 | 1453 (9.5) | 830 (7.0) |
| 5 | 892 (5.8) | 496 (4.2) |
| 6 | 532 (3.5) | 302 (2.6) |
| 7 | 271 (1.8) | 148 (1.3) |
| 8+ | 186 (1.2) | 97 (0.8) |
|  |  |  |
| **Number of 2010 GBD injury types** |  |  |
| **EQ-5D** | **(n=14367)** | **(n=9553)** |
| 1 | 5871 (40.9) | 5871 (40.8) |
| 2 | 3544 (24.7) | 3544 (24.7) |
| 3 | 2064 (14.4) | 2064 (14.4) |
| 4 | 1246 (8.7) | 1246 (8.7) |
| 5 | 707 (4.9) | 707 (4.9) |
| 6 | 412 (2.9) | 412 (2.9) |
| 7 | 251 (1.8) | 251 (1.7) |
| 8+ | 272 (1.9) | 272 (1.9) |
| **GOS-E** | N = 23433 | N = 3686 |
| 1 | 8907 (38.0) | 1147 (31.1) |
| 2 | 5866 (25.0) | 1039 (28.2) |
| 3 | 3589 (15.3) | 676 (18.4) |
| 4 | 2192 (9.4) | 425 (11.5) |
| 5 | 1285 (5.5) | 1094 (5.3) |
| 6 | 736 (3.1) | 97 (2.6) |
| 7 | 422 (1.8) | 61 (1.6) |
| 8+ | 446 (1.9) | 47 (1.3) |
| **SF-12** | N = 15311 | N = 11818 |
| 1 | 5233 (34.2) | 4821 (40.8) |
| 2 | 3834 (25.0) | 3071 (26.0) |
| 3 | 2488 (16.3) | 1777 (15.0) |
| 4 | 1607 (10.5) | 1010 (8.6) |
| 5 | 970 (6.3) | 509 (4.3) |
| 6 | 555 (3.6) | 278 (2.3) |
| 7 | 319 (2.1) | 164 (1.4) |
| 8+ | 305 (2.0) | 188 (1.6) |

**Table S3: Association between number of ICD-10 body regions injured and 6-month disability outcomes**

| **Outcome at 6-months** | **Number of regions injured** | **N** | **n (%) with outcome** | ***ARR (95% CI)** | **p-value** |
| --- | --- | --- | --- | --- | --- |
| Poor recovery (GOS-E<7) | 1 | 9191 | 5362 [58.3] | 1 |  |
|  | 2 | 4381 | 2762 [63.0] | 1.09 [1.06, 1.12] | <0.001 |
|  | 3 | 2381 | 1549 [65.1] | 1.13 [1.09, 1.17] | <0.001 |
|  | 4 | 1607 | 1108 [69.0] | 1.23 [1.18, 1.28] | <0.001 |
|  | 5 | 1052 | 733 [69.7] | 1.23 [1.18, 1.29] | <0.001 |
|  | 6 | 640 | 485 [75.8] | 1.35 [1.28, 1.42] | <0.001 |
|  | 7 | 346 | 274 [79.2] | 1.40 [1.32, 1.49] | <0.001 |
|  | 8+ | 247 | 205 [83.0] | 1.46 [1.37, 1.55] | <0.001 |
| EQ-5D - mobility | 1 | 6109 | 3159 [51.7] | 1 |  |
|  | 2 | 2772 | 1483 [53.5] | 1.09 [1.05, 1.13] | <0.001 |
|  | 3 | 1435 | 690 [48.1] | 1.08 [1.02, 1.14] | 0.01 |
|  | 4 | 985 | 454 [46.1] | 1.16 [1.08, 1.25] | <0.001 |
|  | 5 | 618 | 307 [49.7] | 1.27 [1.17, 1.38] | <0.001 |
|  | 6 | 363 | 181 [49.9] | 1.36 [1.22, 1.52] | <0.001 |
|  | 7 | 201 | 116 [57.7] | 1.64 [1.45, 1.85] | <0.001 |
|  | 8+ | 152 | 100 [65.8] | 1.78 [1.56, 2.03] | <0.001 |
| EQ-5D – self-care | 1 | 6106 | 1927 [31.6] | 1 |  |
|  | 2 | 2766 | 890 [32.2] | 1.09 [1.03, 1.15] | 0.004 |
|  | 3 | 1433 | 387 [27.0] | 1.06 [0.98, 1.15] | 0.18 |
|  | 4 | 984 | 243 [24.7] | 1.16 [1.04, 1.30] | 0.01 |
|  | 5 | 615 | 162 [26.3] | 1.23 [1.08, 1.41] | 0.002 |
|  | 6 | 363 | 99 [27.3] | 1.44 [1.21, 1.71] | <0.001 |
|  | 7 | 201 | 59 [29.4] | 1.67 [1.31, 2.11] | <0.001 |
|  | 8+ | 152 | 64 [42.1] | 2.12 [1.72, 2.62] | <0.001 |
| EQ-5D – usual activities | 1 | 6097 | 3558 [58.4] | 1 |  |
|  | 2 | 2761 | 1724 [62.4] | 1.10 [1.06, 1.14] | <0.001 |
|  | 3 | 1434 | 887 [61.9] | 1.14 [1.09, 1.20] | <0.001 |
|  | 4 | 981 | 602 [61.4] | 1.20 [1.14, 1.27] | <0.001 |
|  | 5 | 614 | 401 [65.3] | 1.28 [1.20, 1.37] | <0.001 |
|  | 6 | 364 | 260 [71.4] | 1.45 [1.34, 1.55] | <0.001 |
|  | 7 | 199 | 147 [73.9] | 1.51 [1.39, 1.65] | <0.001 |
|  | 8+ | 153 | 120 [78.4] | 1.57 [1.43, 1.72] | <0.001 |
|  |  |  |  |  |  |
| EQ-5D – pain/discomfort | 1 | 6047 | 3439 [56.9] | 1 |  |
|  | 2 | 2745 | 1712 [62.4] | 1.12 [1.08, 1.16] | <0.001 |
|  | 3 | 1414 | 914 [64.6] | 1.20 [1.14, 1.25] | <0.001 |
|  | 4 | 973 | 648 [66.6] | 1.28 [1.21, 1.35] | <0.001 |
|  | 5 | 608 | 416 [68.4] | 1.33 [1.25, 1.42] | <0.001 |
|  | 6 | 358 | 264 [73.7] | 1.46 [1.36, 1.57] | <0.001 |
|  | 7 | 200 | 142 [71.0] | 1.41 [1.28, 1.55] | <0.001 |
|  | 8+ | 150 | 120 [80.0] | 1.58 [1.45, 1.73] | <0.001 |
| EQ-5D – anxiety/depression | 1 | 6010 | 2308 [38.4] | 1 |  |
|  | 2 | 2731 | 1202 [44.0] | 1.13 [1.07, 1.19] | <0.001 |
|  | 3 | 1417 | 642 [45.3] | 1.16 [1.08, 1.24] | <0.001 |
|  | 4 | 962 | 436 [45.3] | 1.16 [1.07, 1.26] | <0.001 |
|  | 5 | 606 | 284 [46.9] | 1.19 [1.08, 1.31] | <0.001 |
|  | 6 | 355 | 176 [49.6] | 1.24 [1.10, 1.39] | <0.001 |
|  | 7 | 199 | 100 [50.3] | 1.25 [1.08, 1.45] | 0.004 |
|  | 8+ | 153 | 80 [52.3] | 1.24 [1.06, 1.46] | 0.01 |
|  |  | ***N*** | ***Mean (sd)*** | ****Adjusted difference from single injury group (95% CI)*** | ***p-value*** |
| PCS-12 | 1 | 5387 | 43.1 [11.8] | - |  |
|  | 2 | 2740 | 41.8 [11.8] | -1.8 [-2.3, -1.2] | <0.001 |
|  | 3 | 1571 | 41.2 [11.9] | -2.7 [-3.4, -2.0] | <0.001 |
|  | 4 | 1137 | 40.2 [12.1] | -4.3 [-5.0, -3.5] | <0.001 |
|  | 5 | 737 | 39.8 [12.0] | -4.9 [-5.8, -4.0] | <0.001 |
|  | 6 | 446 | 37.3 [11.6] | -7.7 [-8.9, -6.5] | <0.001 |
|  | 7 | 250 | 35.8 [11.0] | -9.1 [-10.5, -7.6] | <0.001 |
|  | 8+ | 176 | 34.1 [12.0] | -10.9 [-12.6, -9.1] | <0.001 |
|  |  |  |  |  |  |
| MCS-12 | 1 | 5387 | 52.7 [10.5] | - |  |
|  | 2 | 2740 | 51.5 [11.2] | -0.8 [-1.3, -0.3] | 0.002 |
|  | 3 | 1571 | 50.4 [11.7] | -1.4 [-2.0, -0.8] | <0.001 |
|  | 4 | 1137 | 49.7 [11.8] | -1.7 [-2.4, -0.9] | <0.001 |
|  | 5 | 737 | 49.6 [12.2] | -1.6 [-2.5, -0.7] | <0.001 |
|  | 6 | 446 | 48.9 [11.8] | -1.9 [-3.0, -0.8] | 0.001 |
|  | 7 | 250 | 48.6 [12.6] | -2.0 [-3.4, -0.6] | 0.01 |
|  | 8+ | 176 | 48.3 [12.4] | -2.2 [-3.9, -0.6] | 0.01 |

*ARR, Adjusted Relative Risk – adjusted for age and gender of patient, and data source

**Table S4: Association between number of 2010 GBD injury types represented and 6-month disability outcomes**

| **Outcome at 6-months** | **Number of 2010 GBD classifications** | **N** | **n (%) with outcome** | ***ARR (95% CI)** | **p-value** |
| --- | --- | --- | --- | --- | --- |
| Poor recovery (GOS-E<7) | 1 | 7653 | 4453 [58.2] | 1 |  |
|  | 2 | 4928 | 3022 [61.3] | 1.08 [1.05, 1.11] | <0.001 |
|  | 3 | 2965 | 1856 [62.6] | 1.13 [1.09, 1.17] | <0.001 |
|  | 4 | 1825 | 1224 [67.1] | 1.24 [1.19, 1.29] | <0.001 |
|  | 5 | 1073 | 769 [71.7] | 1.34 [1.28, 1.41] | <0.001 |
|  | 6 | 607 | 465 [76.6] | 1.42 [1.35, 1.50] | <0.001 |
|  | 7 | 379 | 314 [82.9] | 1.54 [1.46, 1.63] | <0.001 |
|  | 8+ | 415 | 375 [90.4] | 1.69 [1.62, 1.77] | <0.001 |
| EQ-5D - mobility | 1 | 5186 | 2794 [53.9] | 1 |  |
|  | 2 | 3117 | 1619 [51.9] | 1.06 [1.02, 1.10] | 0.01 |
|  | 3 | 1805 | 805 [44.6] | 1.02 [0.97, 1.08] | 0.41 |
|  | 4 | 1078 | 490 [45.5] | 1.18 [1.10, 1.27] | <0.001 |
|  | 5 | 639 | 289 [45.2] | 1.25 [1.14, 1.37] | <0.001 |
|  | 6 | 363 | 188 [51.8] | 1.47 [1.32, 1.64] | <0.001 |
|  | 7 | 210 | 121 [57.6] | 1.70 [1.50, 1.93] | <0.001 |
|  | 8+ | 237 | 184 [77.6] | 2.40 [2.19, 2.63] | <0.001 |
| EQ-5D – self-care | 1 | 5182 | 1722 [33.2] | 1 |  |
|  | 2 | 3111 | 950 [30.5] | 1.04 [0.98, 1.10] | 0.17 |
|  | 3 | 1805 | 476 [26.4] | 1.05 [0.97, 1.14] | 0.20 |
|  | 4 | 1074 | 261 [24.3] | 1.18 [1.05, 1.32] | 0.004 |
|  | 5 | 639 | 136 [21.3] | 1.13 [0.96, 1.32] | 0.14 |
|  | 6 | 362 | 108 [29.8] | 1.59 [1.35, 1.88] | <0.001 |
|  | 7 | 210 | 70 [33.3] | 1.89 [1.54, 2.32] | <0.001 |
|  | 8+ | 237 | 108 [45.6] | 2.78 [2.34, 3.32] | <0.001 |
|  |  |  |  |  |  |
| EQ-5D – usual activities | 1 | 5175 | 3063 [59.2] | 1 |  |
|  | 2 | 3108 | 1892 [60.9] | 1.08 [1.05, 1.12] | <0.001 |
|  | 3 | 1802 | 1063 [59.0] | 1.12 [1.07, 1.17] | <0.001 |
|  | 4 | 1074 | 658 [61.3] | 1.25 [1.18, 1.32] | <0.001 |
|  | 5 | 638 | 412 [64.6] | 1.36 [1.27, 1.45] | <0.001 |
|  | 6 | 361 | 248 [68.7] | 1.45 [1.34, 1.57] | <0.001 |
|  | 7 | 208 | 160 [76.9] | 1.66 [1.52, 1.81] | <0.001 |
|  | 8+ | 237 | 203 [85.7] | 1.88 [1.76, 2.02] | <0.001 |
|  |  |  |  |  |  |
| EQ-5D – pain/discomfort | 1 | 5129 | 2963 [57.8] | 1 |  |
|  | 2 | 3086 | 1906 [61.8] | 1.11 [1.07, 1.15] | <0.001 |
|  | 3 | 1791 | 1076 [60.1] | 1.13 [1.08, 1.18] | <0.001 |
|  | 4 | 1064 | 692 [65.1] | 1.28 [1.21, 1.35] | <0.001 |
|  | 5 | 626 | 437 [69.8] | 1.40 [1.32, 1.50] | <0.001 |
|  | 6 | 358 | 241 [67.3] | 1.37 [1.27, 1.49] | <0.001 |
|  | 7 | 206 | 152 [73.8] | 1.53 [1.40, 1.68] | <0.001 |
|  | 8+ | 235 | 188 [80.0] | 1.67 [1.54, 1.80] | <0.001 |
| EQ-5D – anxiety/depression | 1 | 5086 | 1946 [38.3] | 1 |  |
|  | 2 | 3082 | 1287 [41.8] | 1.10 [1.04, 1.16] | 0.001 |
|  | 3 | 1786 | 776 [43.5] | 1.14 [1.07, 1.22] | <0.001 |
|  | 4 | 1055 | 491 [46.5] | 1.22 [1.13, 1.32] | <0.001 |
|  | 5 | 626 | 304 [48.6] | 1.25 [1.14, 1.38] | <0.001 |
|  | 6 | 357 | 186 [52.1] | 1.32 [1.17, 1.47] | <0.001 |
|  | 7 | 205 | 110 [53.7] | 1.34 [1.17, 1.54] | <0.001 |
|  | 8+ | 236 | 128 [54.2] | 1.35 [1.18, 1.54] | <0.001 |
|  |  | **N** | **Mean (sd)** | ***Adjusted difference from single injury group (95% CI)** | **p-value** |
| PCS-12 | 1 | 4364 | 42.9 [11.9] | - |  |
|  | 2 | 3114 | 42.3 [11.8] | -1.4 [-2.0, -0.9] | <0.001 |
|  | 3 | 1966 | 42.1 [11.9] | -2.4 [-3.0, -1.7] | <0.001 |
|  | 4 | 1270 | 40.7 [12.2] | -4.5 [-5.3, -3.7] | <0.001 |
|  | 5 | 773 | 39.3 [11.8] | -6.2 [-7.1, -5.2] | <0.001 |
|  | 6 | 434 | 37.9 [11.8] | -7.9 [-9.1, -6.7] | <0.001 |
|  | 7 | 265 | 36.4 [11.0] | -9.8 [-11.3, -8.3] | <0.001 |
|  | 8+ | 258 | 32.7 [10.7] | -13.5 [-15.0, -12.0] | <0.001 |
|  |  |  |  |  |  |
| MCS-12 | 1 | 4364 | 53.0 [10.2] | - |  |
|  | 2 | 3114 | 51.8 [11.2] | -0.9 [-1.4, -0.4] | 0.001 |
|  | 3 | 1966 | 50.4 [11.4] | -1.7 [-2.3, -1.1] | <0.001 |
|  | 4 | 1270 | 49.7 [12.1] | -1.8 [-2.6, -1.1] | <0.001 |
|  | 5 | 773 | 49.5 [12.1] | -1.7 [-2.6, -0.8] | <0.001 |
|  | 6 | 434 | 48.6 [12.4] | -2.3 [-3.5, -1.2] | <0.001 |
|  | 7 | 265 | 49.0 [12.0] | -1.6 [-3.0, -0.2] | 0.03 |
|  | 8+ | 258 | 48.4 [12.6] | -1.9 [-3.3, -0.4] | 0.01 |

*ARR, Adjusted Relative Risk – adjusted for age and gender of patient, and data source

**Table S5: Association between number of ICD-10 body regions injured and 24-month disability outcomes**

| **Outcome at 24-months** | **Number of regions injured** | **N** | **n (%) with outcome** | ***ARR (95% CI)** | **p-value** |
| --- | --- | --- | --- | --- | --- |
| Poor recovery (GOS-E<7) | 1 | 1093 | 638 [58.4] | 1 |  |
|  | 2 | 885 | 556 [62.8] | 1.11 [1.03, 1.18] | 0.003 |
|  | 3 | 767 | 460 [60.0] | 1.09 [1.01, 1.18] | 0.02 |
|  | 4 | 596 | 344 [57.7] | 1.10 [1.01, 1.19] | 0.03 |
|  | 5 | 480 | 313 [65.2] | 1.24 [1.14, 1.35] | <0.001 |
|  | 6 | 353 | 254 [72.0] | 1.38 [1.27, 1.50] | <0.001 |
|  | 7 | 199 | 152 [76.4] | 1.48 [1.35, 1.63] | <0.001 |
|  | 8+ | 154 | 726 [81.8] | 1.55 [1.42, 1.71] | <0.001 |
| EQ-5D - mobility | 1 | 1146 | 342 [29.8] | 1 |  |
|  | 2 | 741 | 237 [32.0] | 1.05 [0.93, 1.18] | 0.46 |
|  | 3 | 619 | 224 [36.2] | 1.17 [1.03, 1.33] | 0.01 |
|  | 4 | 486 | 173 [35.6] | 1.29 [1.12, 1.49] | 0.001 |
|  | 5 | 392 | 172 [43.9] | 1.55 [1.35, 1.78] | <0.001 |
|  | 6 | 277 | 119 [43.0] | 1.56 [1.32, 1.84] | <0.001 |
|  | 7 | 155 | 82 [52.9] | 1.96 [1.66, 2.33] | <0.001 |
|  | 8+ | 120 | 73 [60.8] | 2.03 [1.72, 2.39] | <0.001 |
| EQ-5D – self-care | 1 | 1147 | 192 [16.7] | 1 |  |
|  | 2 | 740 | 139 [18.8] | 1.05 [0.88, 1.25] | 0.62 |
|  | 3 | 619 | 136 [22.0] | 1.20 [1.00, 1.44] | 0.05 |
|  | 4 | 484 | 82 [16.9] | 1.06 [0.85, 1.34] | 0.59 |
|  | 5 | 391 | 77 [16.7] | 1.19 [0.95, 1.49] | 0.13 |
|  | 6 | 277 | 82 [29.6] | 1.86 [1.48, 2.32] | <0.001 |
|  | 7 | 155 | 47 [30.3] | 1.95 [1.49, 2.55] | <0.001 |
|  | 8+ | 119 | 46 [38.7] | 2.18 [1.68, 2.83] | <0.001 |
| EQ-5D – usual activities | 1 | 1146 | 404 [35.3] | 1 |  |
|  | 2 | 741 | 326 [44.0] | 1.15 [1.04, 1.28] | 0.01 |
|  | 3 | 619 | 294 [47.5] | 1.20 [1.08, 1.34] | 0.001 |
|  | 4 | 484 | 233 [48.1] | 1.27 [1.13, 1.43] | <0.001 |
|  | 5 | 392 | 237 [60.5] | 1.58 [1.41, 1.76] | <0.001 |
|  | 6 | 276 | 162 [58.7] | 1.53 [1.34, 1.74] | <0.001 |
|  | 7 | 154 | 105 [68.2] | 1.80 [1.57, 2.07] | <0.001 |
|  | 8+ | 120 | 86 [71.7] | 1.79 [1.56, 2.04] | <0.001 |
|  |  |  |  |  |  |
| EQ-5D – pain/discomfort | 1 | 1138 | 468 [41.1] | 1 |  |
|  | 2 | 730 | 343 [47.0] | 1.17 [1.05, 1.29] | 0.004 |
|  | 3 | 611 | 339 [55.5] | 1.38 [1.24, 1.53] | <0.001 |
|  | 4 | 481 | 262 [54.5] | 1.38 [1.23, 1.54] | <0.001 |
|  | 5 | 392 | 240 [61.2] | 1.56 [1.40, 1.74] | <0.001 |
|  | 6 | 275 | 181 [65.8] | 1.67 [1.49, 1.88] | <0.001 |
|  | 7 | 153 | 105 [68.6] | 1.76 [1.54, 2.01] | <0.001 |
|  | 8+ | 119 | 90 [75.6] | 1.87 [1.64, 2.13] | <0.001 |
|  |  |  |  |  |  |
| EQ-5D – anxiety/depression | 1 | 1139 | 356 [31.3] | 1 |  |
|  | 2 | 735 | 282 [38.4] | 1.11 [0.98, 1.25] | 0.12 |
|  | 3 | 610 | 244 [40.0] | 1.10 [0.97, 1.26] | 0.13 |
|  | 4 | 478 | 201 [42.1] | 1.14 [0.99, 1.31] | 0.07 |
|  | 5 | 388 | 173 [44.6] | 1.21 [1.05, 1.39] | 0.01 |
|  | 6 | 270 | 133 [49.3] | 1.31 [1.13, 1.53] | <0.001 |
|  | 7 | 152 | 78 [51.3] | 1.36 [1.14, 1.63] | 0.001 |
|  | 8+ | 118 | 72 [61.0] | 1.59 [1.34, 1.89] | <0.001 |
|  |  | **N** | **Mean (sd)** | ***Adjusted difference from single injury group (95% CI)** | **p-value** |
| PCS-12 | 1 | 539 | 47.5 [11.3] | - |  |
|  | 2 | 466 | 45.8 [11.8] | -1.9 [-3.3, -0.4] | 0.01 |
|  | 3 | 480 | 44.3 [12.4] | -3.3 [-4.7, -1.8] | <0.001 |
|  | 4 | 396 | 43.6 [12.8] | -4.3 [-5.8, -2.8] | <0.001 |
|  | 5 | 321 | 41.4 [12.0] | -6.7 [-8.3, -5.1] | <0.001 |
|  | 6 | 209 | 39.7 [12.6] | -8.3 [-10.1, -6.4] | <0.001 |
|  | 7 | 124 | 38.5 [12.4] | -9.8 [-12.1, -7.5] | <0.001 |
|  | 8+ | 108 | 34.7 [11.4] | -13.1 [-15.5, -10.6] | <0.001 |
|  |  |  |  |  |  |
| MCS-12 | 1 | 539 | 51.4 [10.9] | - |  |
|  | 2 | 466 | 51.0 [11.4] | -0.3 [-1.7, 1.1] | 0.67 |
|  | 3 | 480 | 49.5 [12.2] | -1.8 [-3.2, -0.3] | 0.02 |
|  | 4 | 396 | 50.3 [11.2] | -0.8 [-2.3, 0.7] | 0.32 |
|  | 5 | 321 | 49.5 [11.5] | -1.7 [-3.2, -0.1] | 0.04 |
|  | 6 | 209 | 48.4 [12.0] | -2.4 [-4.2, -0.6] | 0.01 |
|  | 7 | 124 | 48.8 [13.2] | -2.1 [-4.3, 0.2] | 0.07 |
|  | 8+ | 108 | 47.2 [11.5] | -3.5 [-5.9, -1.1] | 0.004 |

*ARR, Adjusted Relative Risk – adjusted for age and gender of patient, and data source

**Table S6: Association between number of 2010 GBD injury types represented and 24-month disability outcomes**

| **Outcome at 24-months** | **Number of 2010 GBD classifications** | **N** | **n (%) with outcome** | ***ARR (95% CI)** | **p-value** |
| --- | --- | --- | --- | --- | --- |
| Poor recovery (GOS-E<7) | 1 | 561 | 327 [58.3] | 1 |  |
|  | 2 | 835 | 527 [63.1] | 1.14 [1.06, 1.24] | 0.001 |
|  | 3 | 869 | 484 [55.7] | 1.05 [0.96, 1.15] | 0.26 |
|  | 4 | 771 | 451 [58.5] | 1.16 [1.06, 1.27] | 0.001 |
|  | 5 | 563 | 363 [64.5] | 1.32 [1.20, 1.44] | <0.001 |
|  | 6 | 376 | 260 [69.2] | 1.39 [1.26, 1.54] | <0.001 |
|  | 7 | 253 | 187 [73.9] | 1.50 [1.36, 1.66] | <0.001 |
|  | 8+ | 299 | 244 [81.6] | 1.69 [1.54, 1.85] | <0.001 |
| EQ-5D - mobility | 1 | 731 | 218 [29.8] | 1 |  |
|  | 2 | 733 | 230 [31.4] | 1.01 [0.88, 1.16] | 0.90 |
|  | 3 | 684 | 225 [32.9] | 1.10 [0.95, 1.27] | 0.22 |
|  | 4 | 605 | 215 [35.5] | 1.23 [1.06, 1.42] | 0.01 |
|  | 5 | 457 | 171 [37.4] | 1.39 [1.18, 1.63] | <0.001 |
|  | 6 | 300 | 121 [40.3] | 1.45 [1.22, 1.72] | <0.001 |
|  | 7 | 195 | 101 [51.8] | 1.93 [1.62, 2.31] | <0.001 |
|  | 8+ | 231 | 141 [61.0] | 2.34 [2.00, 2.73] | <0.001 |
| EQ-5D – self-care | 1 | 732 | 120 [16.4] | 1 |  |
|  | 2 | 732 | 143 [19.5] | 1.05 [0.86, 1.28] | 0.62 |
|  | 3 | 683 | 111 [16.3] | 0.88 [0.71, 1.09] | 0.25 |
|  | 4 | 605 | 119 [19.7] | 1.12 [0.90, 1.39] | 0.32 |
|  | 5 | 457 | 90 [19.7] | 1.23 [0.97, 1.56] | 0.09 |
|  | 6 | 299 | 76 [25.4] | 1.52 [1.19, 1.94] | 0.001 |
|  | 7 | 194 | 57 [29.4] | 1.85 [1.43, 2.41] | <0.001 |
|  | 8+ | 230 | 85 [37.0] | 2.42 [1.92, 3.05] | <0.001 |
|  |  |  |  |  |  |
| EQ-5D – usual activities | 1 | 731 | 248 [33.9] | 1 |  |
|  | 2 | 732 | 286 [39.1] | 1.02 [0.90, 1.16] | 0.74 |
|  | 3 | 684 | 307 [44.9] | 1.13 [1.00, 1.28] | 0.06 |
|  | 4 | 605 | 302 [49.9] | 1.27 [1.12, 1.44] | <0.001 |
|  | 5 | 457 | 241 [52.7] | 1.38 [1.21, 1.57] | <0.001 |
|  | 6 | 298 | 169 [56.7] | 1.45 [1.26, 1.67] | <0.001 |
|  | 7 | 195 | 126 [64.6] | 1.69 [1.46, 1.96] | <0.001 |
|  | 8+ | 230 | 168 [73.0] | 1.93 [1.70, 2.20] | <0.001 |
|  |  |  |  |  |  |
| EQ-5D – pain/discomfort | 1 | 726 | 310 [42.7] | 1 |  |
|  | 2 | 727 | 325 [44.7] | 1.10 [0.97, 1.23] | 0.13 |
|  | 3 | 669 | 317 [47.4] | 1.21 [1.07, 1.37] | 0.003 |
|  | 4 | 599 | 331 [55.3] | 1.41 [1.25, 1.60] | <0.001 |
|  | 5 | 457 | 266 [58.2] | 1.50 [1.32, 1.71] | <0.001 |
|  | 6 | 299 | 188 [62.9] | 1.62 [1.41, 1.85] | <0.001 |
|  | 7 | 194 | 122 [62.9] | 1.62 [1.39, 1.88] | <0.001 |
|  | 8+ | 228 | 169 [74.1] | 1.90 [1.67, 2.16] | <0.001 |
|  |  |  |  |  |  |
| EQ-5D – anxiety/depression | 1 | 727 | 185 [25.5] | 1 |  |
|  | 2 | 728 | 264 [37.3] | 1.28 [1.09, 1.50] | 0.003 |
|  | 3 | 676 | 272 [39.5] | 1.26 [1.07, 1.48] | 0.01 |
|  | 4 | 594 | 238 [39.9] | 1.28 [1.08, 1.51] | 0.004 |
|  | 5 | 453 | 218 [47.4] | 1.50 [1.26, 1.77] | <0.001 |
|  | 6 | 295 | 134 [45.1] | 1.41 [1.17, 1.69] | <0.001 |
|  | 7 | 190 | 99 [52.4] | 1.64 [1.35, 1.99] | <0.001 |
|  | 8+ | 227 | 129 [56.6] | 1.74 [1.46, 2.08] | <0.001 |
|  |  | **N** | **Mean (sd)** | ***Adjusted difference from single injury group (95% CI)** | **p-value** |
| PCS-12 | 1 | 235 | 46.6 [12.1] | - |  |
|  | 2 | 433 | 46.7 [11.5] | -0.2 [-2.1, 1.6] | 0.82 |
|  | 3 | 496 | 46.6 [11.4] | -0.5 [-2.4, 1.3] | 0.56 |
|  | 4 | 504 | 44.3 [12.4] | -3.0 [-4.8, -1.1] | 0.002 |
|  | 5 | 380 | 41.7 [12.9] | -5.7 [-7.6, -3.8] | <0.001 |
|  | 6 | 247 | 40.8 [11.8] | -6.7 [-8.8, -4.6] | <0.001 |
|  | 7 | 161 | 39.7 [12.4] | -8.0 [-10.4, -5.6] | <0.001 |
|  | 8+ | 187 | 35.6 [12.1] | -11.7 [-14.0, -9.4] | <0.001 |
|  |  |  |  |  |  |
| MCS-12 | 1 | 235 | 52.6 [10.3] | - |  |
|  | 2 | 433 | 50.9 [11.2] | -1.3 [-3.1, 0.6] | 0.18 |
|  | 3 | 496 | 50.2 [11.6] | -2.0 [-3.7, -0.2] | 0.03 |
|  | 4 | 504 | 49.8 [11.9] | -2.1 [-3.9, -0.3] | 0.02 |
|  | 5 | 380 | 49.7 [11.7] | -2.2 [-4.1, -0.4] | 0.02 |
|  | 6 | 247 | 49.0 [11.6] | -3.1 [-5.1, -1.0] | 0.004 |
|  | 7 | 161 | 48.5 [12.2] | -3.3 [-5.6, -1.0] | 0.01 |
|  | 8+ | 187 | 48.5 [12.1] | -2.9 [-5.1, -0.7] | 0.01 |

*ARR, Adjusted Relative Risk – adjusted for age and gender of patient,
